# Hereditary Ovarian Cancer: Not Only *BRCA* 1 and 2 Genes

**DOI:** 10.1155/2015/341723

**Published:** 2015-05-17

**Authors:** Angela Toss, Chiara Tomasello, Elisabetta Razzaboni, Giannina Contu, Giovanni Grandi, Angelo Cagnacci, Russell J. Schilder, Laura Cortesi

**Affiliations:** ^1^Department of Oncology, Haematology and Respiratory Diseases, University Hospital of Modena, 41124 Modena, Italy; ^2^Department of Obstetrics Gynecology and Pediatrics, Obstetrics and Gynecology Unit, University Hospital of Modena, 41124 Modena, Italy; ^3^Department of Medical Oncology, Kimmel Cancer Center, Thomas Jefferson University Hospital, Philadelphia, PA 19107, USA

## Abstract

More than one-fifth of ovarian tumors have hereditary susceptibility and, in about 65–85% of these cases, the genetic abnormality is a germline mutation in *BRCA* genes. Nevertheless, several other suppressor genes and oncogenes have been associated with hereditary ovarian cancers, including the mismatch repair (*MMR*) genes in Lynch syndrome, the tumor suppressor gene, *TP53*, in the Li-Fraumeni syndrome, and several other genes involved in the double-strand breaks repair system, such as *CHEK2, RAD51, BRIP1*, and *PALB2*. The study of genetic discriminators and deregulated pathways involved in hereditary ovarian syndromes is relevant for the future development of molecular diagnostic strategies and targeted therapeutic approaches. The recent development and implementation of next-generation sequencing technologies have provided the opportunity to simultaneously analyze multiple cancer susceptibility genes, reduce the delay and costs, and optimize the molecular diagnosis of hereditary tumors. Particularly, the identification of mutations in ovarian cancer susceptibility genes in healthy women may result in a more personalized cancer risk management with tailored clinical and radiological surveillance, chemopreventive approaches, and/or prophylactic surgeries. On the other hand, for ovarian cancer patients, the identification of mutations may provide potential targets for biologic agents and guide treatment decision-making.

## 1. Introduction

Ovarian cancer represents the leading cause of cancer deaths among gynecological malignancies, accounting worldwide for about 225.000 new cancer cases (3.7% of all female cancers) and about 140,000 deaths (4.2% of all deaths in women) every year [1]. In the United States, 21,980 estimated new cases and 14,270 estimated deaths were expected in 2014 [2]. The lack of effective early detection strategies and an unfavorable anatomy are associated with the advanced stage at diagnosis and poor prognosis often found in ovarian cancer patients. Therefore, overall survival (OS) is the worst among all of the gynecologic malignancies, with a five-year relative survival rate of 44% for all stages in all races [2].

**Figure 1:**
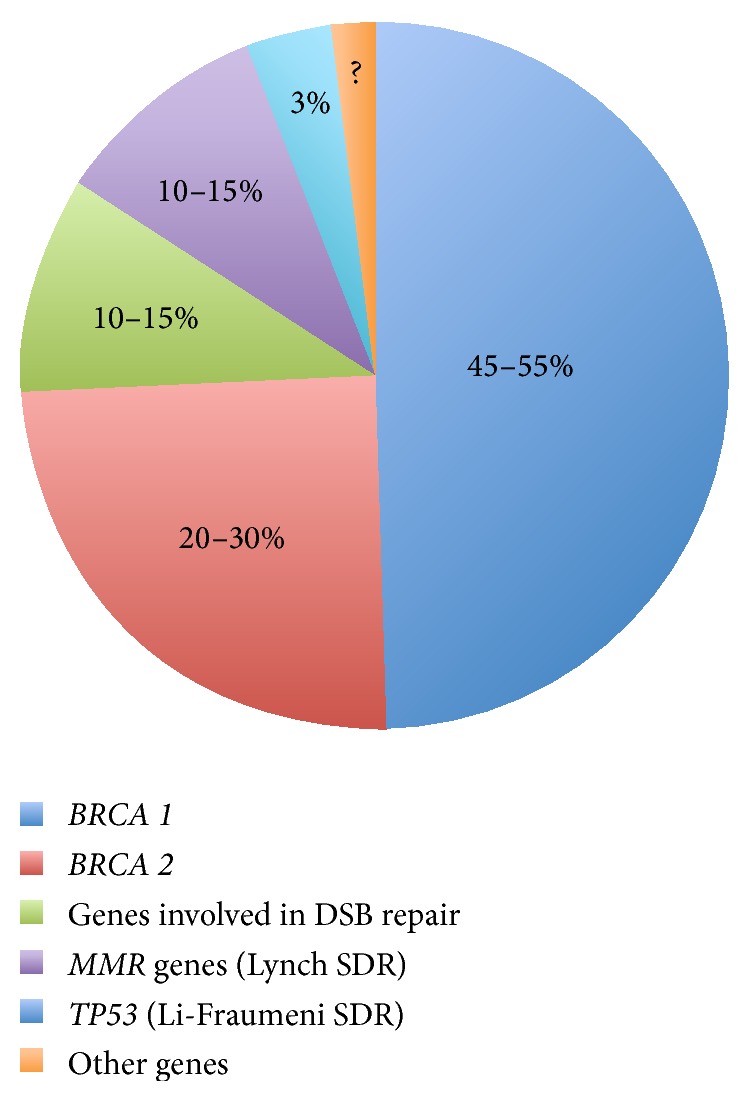
Susceptibility genes and their prevalence in hereditary ovarian syndromes.

More than one-fifth (about 23%) of ovarian carcinomas have been related to hereditary conditions [3]. Particularly, in about 65–85% of hereditary ovarian tumors, the genetic abnormality is a germline mutation in* BRCA *genes that causes DNA repair defects.* BRCA1* and* BRCA2* mutation carriers have an increased lifetime risk of developing breast and ovarian cancer (up to 85% for breast cancer and up to 54% for ovarian cancer), as well as pancreatic and prostate cancer [4–8]. Nevertheless, several other suppressor genes and oncogenes have been associated with hereditary ovarian cancer (i.e.,* TP53*,* BARD1*,* CHEK2*,* RAD51*, and* PALB2* [3, 9–11]). To date, at least 16 genes are known to be involved in the mechanism of hereditary ovarian tumorigenesis and several other mutations remain unknown and cannot be detected by specific tests [12] (Figure 1).

The identification of a mutation in ovarian cancer susceptibility genes represents a fundamental step in the diagnosis and treatment of these tumors. First of all, the detection of a specific mutated gene in healthy women may justify more intensive and personalized surveillance programs, chemopreventive approaches, and/or prophylactic surgery that would not otherwise be justified by family history alone. Moreover, the identification of a mutation in already affected patients may provide fundamental knowledge of the pathogenesis of their tumors. Therefore, this genetic evaluation at diagnosis may help to identify potential targets for specific drugs, that is, PARP inhibitors and alkylating agents, and to guide decision-making on treatment strategies. In this context, next-generation sequencing (NGS) technologies have recently provided an unprecedented opportunity to simultaneously analyze multiple cancer susceptibility genes, reduce delays and costs, and optimize the molecular diagnosis of hereditary ovarian cancer.

We reviewed the available published data regarding the molecular pathways involved in the pathogenesis of non-*BRCA* hereditary ovarian cancer and the possible applications of NGS technologies in these hereditary cancer syndromes.

## 2. Clinical, Histopathological, and Molecular Features of Ovarian Cancer

Ovarian cancer is a heterogeneous disease that includes different biological behaviors at the clinical and molecular level. More than 90% of ovarian cancers are epithelial, while about 10% of ovarian cancers develop from germ cells or granulosa-theca cells. Epithelial tumors may arise from the ovarian surface epithelium but also may arise from fallopian tube, foci of endometriosis, or the peritoneum [13]. Four main histotypes characterize epithelial ovarian cancer: serous, endometrioid, mucinous, and clear cell. Interestingly, each histotype shows patterns of gene expression that correlate with their morphological counterparts in normal tissues. Particularly, alterations in serous tumors correlated with those in normal fallopian tube, mucinous cancers correlated with those in normal colonic mucosa, and both endometrioid and clear cell histotypes correlated with changes in normal endometrium [14].

Two novel hypotheses for the pathogenesis of serous ovarian cancer have been recently proposed. According to the first mechanism, precursors of ovarian cancer develop in the fimbria from occult serous tubal intraepithelial carcinoma (STIC), and only subsequently do they involve the ovary. The second theory supports the implantation of normal epithelium from the fimbria onto the ovarian surface during ovulation, which results in a cortical inclusion cyst (CICs) where malignant transformation can arise [15, 16].

At the molecular level, sporadic ovarian cancer is characterized by wide genetic instability linked to the modulation of several genes. According to clinical behavior and the molecular genetic abnormalities, ovarian cancer can be classified into two different types (Table 1). Type I tumors include low-grade serous carcinomas, borderline serous tumors, low-grade endometrioid, and mucinous and clear-cell carcinomas [15, 17]. These kinds of tumors are relatively genetically stable, and the most frequent mutations involve* KRAS*,* BRAF*,* ERBB2*,* PTEN*,* PIK3CA*, b-catenin gene (*CTNNB1*),* ARID1A*, and* PPP2R1A*. On the other hand, type II ovarian cancers include high-grade serous carcinomas, carcinosarcomas, and undifferentiated cancers. Type II tumors, which comprise almost 70% of all epithelial tumors, are aggressive and present in advanced stages. At the time of presentation, they exhibit high genomic instability and, in up to 95% of patients, the gene mutated is* TP53*. Moreover, this type of tumor is characteristic of* BRCA1* and* BRCA2* mutation carriers and mostly arises from STICs [15, 17].

## 3. Mismatch Repair Genes and Lynch Syndrome

In the mid1960s, Lynch and colleagues described an autosomal-dominant hereditary syndrome that predisposes young people (mean age 45 years) not affected by adenomatous colonic polyps, to develop colorectal cancer with predilection proximal to the splenic flexure [18, 19]. Subsequent publications on the syndrome reported that members of these families were also prone to excesses of extracolonic cancers, including carcinomas of the endometrium, ovary, stomach, small bowel, hepatobiliary tract, pancreas, renal pelvis, ureter, breast, prostate, and brain (particularly glioblastomas) [20–22].

Lynch syndrome (LS), also known as hereditary nonpolyposis colon cancer (HNPCC), accounts for 10–15% of all hereditary ovarian cancers [23]. The cumulative lifetime risk of ovarian cancer is estimated to be 6–10% in proven or probable* MSH2* and* MLH1* mutation carriers, with an average age of onset of 51 years in families associated with* MLH1* mutations and 45 years in families associated with* MSH2* mutations [24–26]. From the clinical point of view, ovarian cancers in LS are mostly endometrioid or clear cell [27–31], and the tumors are less advanced at the time of diagnosis, showing strikingly high stage-specific survival rates [24, 32, 33]. In particular, Vierkoetter and coinvestigators found that patients under the age of 53 with clear cell or endometrioid ovarian carcinomas are at a clinically significant risk for loss of mismatch repair (MMR) expression and LS, suggesting that routine screening with immunohistochemical staining in these patients should be considered [34].

**Figure 2:**
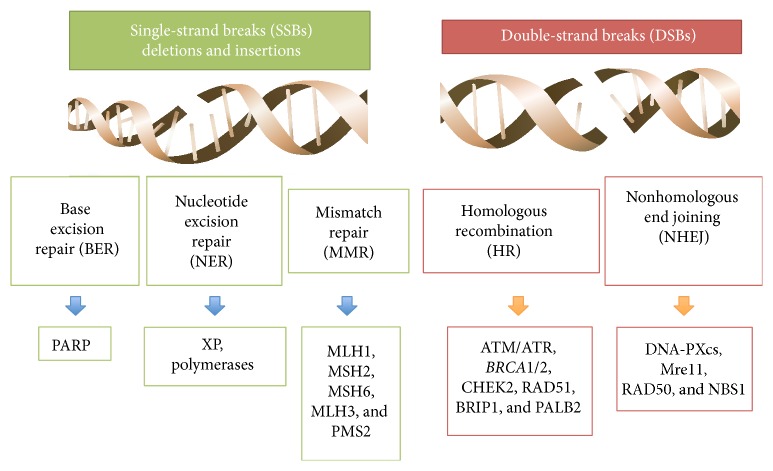
Type of DNA damage, repair pathways, and repair enzymes involved in each pathway.

LS is determined by germline mutations in MMR genes (*MLH1*,* MSH2*,* MSH6*,* MLH3*, and* PMS2*), which lead to the loss of expression of one of the MMR proteins. In LS, one mutated allele of an MMR gene is inherited, and the loss of the second allele occurs somatically, due to mutation, methylation, or a combination of both. The rare case where both inherited alleles are mutated is called “the constitutional MMR deficiency syndrome” and leads to cancer during childhood [35]. The mismatch repair (MMR) system, together with the base excision repair (BER) and the nucleic acid excision repair (NER) system, is the DNA repair mechanism employed to remove single-strand breaks. On the other hand, double-strand breaks are corrected by the homologous recombination (HR) and nonhomologous end joining (NHEJ) (Figure 2) [8]. Failure of the MMR system, which occurs in families with LS, results in the accumulation of repeated nucleotide sequences phenotypically expressed as microsatellite instability (MSI). Microsatellites are short tandem (1–6 base pairs) repeated DNA sequences with high susceptibility for replication errors. Several oncogenes and tumor suppressor genes contain microsatellites, including* TGFβR2*,* IGFIIR*,* BAX*, and DNA double-strand break (DSB) repair genes, such as* Mre11* and* RAD50*. Consequently, impairment of MMR could cause mutations in many genes implicated in tumorigenesis [36–40].

Nevertheless, carcinogenesis in ovarian cancers associated with LS has not been completely explained beyond the description of MMR defects. Recently, Niskakoski and colleagues [41] investigated 107 ovarian tumors (20 from LS and 87 sporadic), in order to gain insights into ovarian tumorigenesis and to analyze molecular alterations that differ between LS and sporadic ovarian cancer. As previously described, LS-associated ovarian cancers were more likely at diagnosis to be of low-grade, early stage, early onset and generally had an overall better prognosis. All 20 LS-associated ovarian carcinomas did not show mutations on* TP53*,* KRAS* (exon 2), or* BRAF* (V600E), while, in sporadic ovarian carcinomas,* TP53* was often abnormal (overexpressed or completely missing) and* KRAS* (exon 2) mutations occurred with a frequency of 8%. In fact, overexpression of* TP53* in ovarian cancer has been linked with poor prognosis, high grade histology, and advanced stages at diagnosis [42]. Interestingly, the frequencies of* PIK3CA* mutations were similar in endometrioid and clear cell carcinomas from LS patients (32%) compared to their sporadic counterparts (36%). The frequency of* PIK3CA* mutations in LS-associated ovarian carcinomas is in accordance with the reported high survival, since recent evidence suggests that* PIK3CA* mutations and the PI3K/AKT pathway activation are associated with a favorable prognosis in ovarian cancer [43]. These data showed that ovarian cancers seem to resemble colorectal cancers from LS carriers, which are associated with higher stage-specific survival [44] and fewer* TP53* and* BRAF* abnormalities compared to sporadic tumors [45] and* PIK3CA* mutations in about 20% of cases [46]. Furthermore, Niskakoski and colleagues [41] found differences in the analysis of cyclin-dependent kinase inhibitor 2B (*CDKN2B*) and long interspersed nucleotide element 1 (*LINE1*). Hypomethylation of* CDKN2B* and* LINE1* was significantly increased in sporadic ovarian cancers compared with LS cases. Since* LINE1* plays an important role in advanced stages of ovarian cancer, this result is consistent with better prognosis showed by LS-associated ovarian cancer [47].

More recently, Jönsson and colleagues [48] performed a global gene expression analysis on 24 LS-associated and 24 sporadic ovarian tumors, with the aim of identifying gene expression profiles and genetic discriminators associated with LS. The most frequently upregulated genes in LS included* PTPRH*,* BIRC3*,* SHH*, and* TNFRSF6B*. The genes involved were predominantly related to cell growth, proliferation, and cell-to-cell signaling and interaction. On the other hand, immunohistochemical staining showed positivity for p-mTOR in 60% of LS tumors, EGFR in 30% of LS tumors, and loss of PTEN in 74% of LS tumors. Moreover, mutations in* KRAS* and* BRAF*, which may activate the mTOR/PI3K/AKT pathway, are common in low-grade ovarian cancers (60%) [49, 50].

The study of genetic discriminators and deregulated pathways involved in LS-associated ovarian tumorigenesis may be relevant for the future development of molecular diagnostics and targeted therapeutics. Unfortunately, given the relative rarity of LS-associated hereditary ovarian cancer, the sample sizes in these studies were small and confirmation in a larger series is necessary. Moreover, other common limitations of these studies may be related to the techniques used for the analyses, including immunohistochemistry and genetic testing on paraffin-embedded tumor tissues.

## 4. *TP53* and Li-Fraumeni Syndrome

The Li-Fraumeni syndrome (LFS) is an autosomal dominant cancer syndrome determined by heterozygous germline mutations in the tumor suppressor gene* TP53* (chromosome 17p13).* TP53* codes for a transcription factor activated in response to various stress signals and implicated in cell proliferation, apoptosis, and genomic stability. It represents the most frequently mutated gene in human cancer, with the highest prevalence of acquired mutations in epithelial ovarian (47%), colorectal (43%), head/neck (42%), and esophageal (41%) cancers [51, 52]. Because of its comprehensive role as a cancer suppressor gene,* TP53* is also defined as the “guardian of the genome” [53]. The most common mutation observed in germline and sporadic cases is the missense mutation (about 75%), resulting in a defective transcriptional activity. Tumors developed from acquired* TP53* mutations are characterized by worse survival rates, increased resistance to chemotherapy and radiation, and elevated relapse rates [54–56].

Li and Fraumeni Jr. proposed for the first time in 1969 the theory of a hereditary cancer syndrome characterized by the early development of multiple tumors [57]. Particularly, about 50% of patients with LFS develop the first tumor by age of 30 [58, 59], and almost one-third (15–35%) of them will develop multiple primary cancers over their lifetimes [60, 61]. Breast cancer, sarcoma and brain, and adrenocortical carcinoma represent about 77–80% of LFS-associated tumors. Less frequent malignancies associated with LS include leukemia and lung, colorectal, skin, gastric, and ovarian cancer and these account for 15% of the tumors [62]. Nevertheless, these less frequent tumors are present in the general population; thus, their presence in LFS families could be because of chance. On the other hand, in the context of a germline* TP53* mutation, these tumors occur at earlier than expected median age at diagnosis. Particularly, for ovarian cancer the median age is 39.5, compared with 64.3 years for sporadic cases [63]. The lack of comprehensive studies regarding the genetic pathways involved in LFS-associated ovarian tumorigenesis is principally due to the extreme rarity of this syndrome in the general population.

## 5. Genes Involved in Double-Strand Breaks Repair

**Figure 3:**
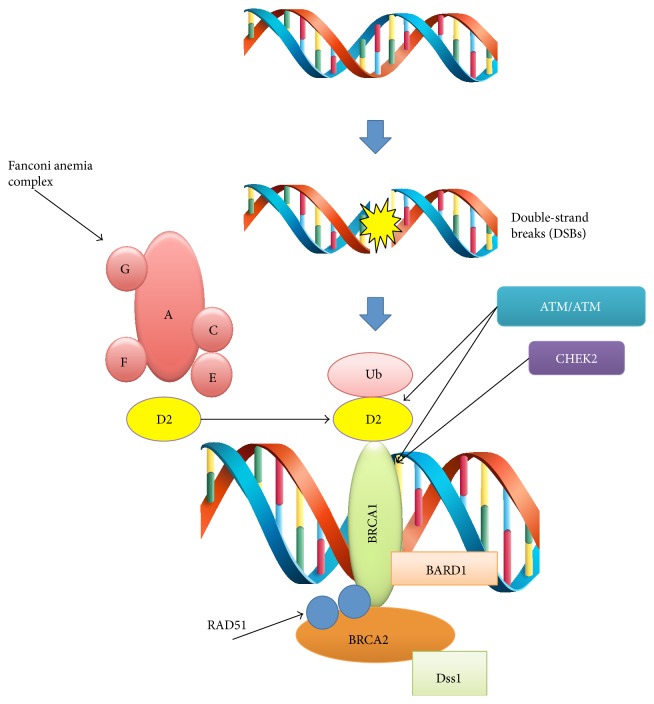
Proteins involved in the homologous recombination (HR) system.

As previously described, double-strand breaks (DSBs) are repaired by homologous recombination (HR) and nonhomologous end joining (NHEJ) (Figure 2). HR provides accurate recombination using a sister chromatid as a template which maintains genomic stability. Several proteins are widely involved in the HR system, including BRCA1/2, ATM, CHEK2, RAD51, and Fanconi anemia proteins (BRIP1 [64] and PALB2 [65]) (Figure 3) [66]. Particularly, DSBs activate the kinases ATM, ATR, and CHEK2, which in turn phosphorylate BRCA1, modulating its function. The role of BRCA1 in DNA repair and in cell cycle regulation is to cause G1-S, S, or G2-M phase arrest depending on the residues phosphorylated. BRCA1 forms a complex with BARD1, a protein with structural similarity, which is important for BRCA1 stability. More recently, the BRCA1-BARD1 complex has been found to play a role in ubiquitination and degradation of RNA polymerase II, inhibiting transcription and RNA processing, in order to eliminate prematurely terminated transcripts and clear the damaged DNA region for the intervention of DNA repair enzymes. In parallel, BRCA2 participates in the repair of DSBs modulating the recombinase function of RAD51. BRCA2 is necessary for the transport of RAD51 into the nucleus and to the site of DNA damage, where RAD51 is released to form the nucleoprotein filament required for recombination [67–69].

**Figure 4:**
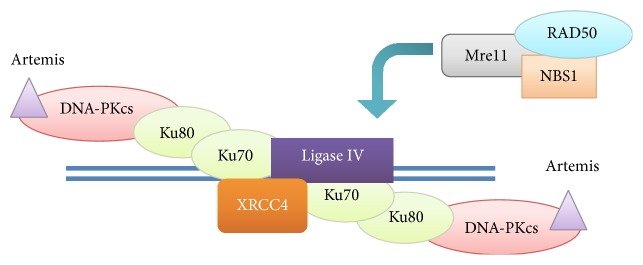
Proteins involved in the nonhomologous end joining (NHEJ).

On the other hand, although less accurate, NHEJ plays a crucial role in minimizing DNA damage in both G0 and G1 phases of the cell cycle, when HR cannot be supplied. However, when a defect occurs in one of the enzymes involved in HR, the DSBs are repaired by error prone mechanisms, mostly NHEJ, resulting in an increased risk of new chromosomal defects, and, therefore, the development of cancer [70]. In the first step of NHEJ, the heterodimer Ku70/Ku80 breaks the DNA ends and improves the stability of the NHEJ related enzymes at the DNA termini. Two Ku70/Ku80 heterodimers recruit DNA-dependent protein kinases (DNA-PKcs) to the DNA ends. The resulting complex of DNA-PKcs and its substrate, Artemis, have shown an endonuclease activity; therefore, it processes the DNA termini in order to prepare them for the intervention of XRCC4-Ligase IV. The nuclease functions of Artemis seem to be accomplished by the complex of RAD50, Mre11, and NBS1, where in vitro models have shown to interact also with Ligase IV and Ku homologues (Figure 4) [71].

As mentioned above, several proteins interact and cooperate with BRCA1 and BRCA2 proteins in the DNA repair process, and, therefore, in the maintenance of genomic stability. It has been hypothesized that genes coding for these proteins would be alternative candidates for ovarian cancer susceptibility. Particularly, tumors with a defect in the HR system other than* BRCA* express the BRCAness profile. These tumors present a specific phenotype with features and behavior similar to* BRCA*-related ovarian cancers [8], including sensitivity to DNA-damaging agents (i.e., platinum), improved disease-free intervals and survival rates, and high-grade serous histology [72, 73]. Interestingly, these BRCAness patients are at increased risk for both ovarian and breast cancers, similar to* BRCA* carriers. The main genes involved in the BRCAness syndrome in ovarian cancer are listed below.

### 5.1. *RAD51*

Loveday and colleagues [74] identified truncating* RAD51D* mutations in 8 of 911 familial breast-ovarian cancer pedigrees, demonstrating that* RAD51D* mutations confer a sixfold increased risk of ovarian cancer but cause only a small increase in breast cancer. Similarly, Meindl and associates [75] analyzed 1,100 German families with gynecological malignancies and identified 6 monoallelic pathogenic mutations in* RAD51C* that confer an increased risk for both breast and ovarian cancers. Finally, Blanco and colleagues [76] recently screened a large series of 516* BRCA1/BRCA2*-negative patients from breast and/or ovarian cancer families for* RAD51C* mutations and identified 3 germline pathogenic mutations. These results confirmed that* RAD51C* contributes to ovarian cancer susceptibility in families with breast and ovarian cancer cases.

### 5.2. *PALB2*

*PALB2* mutations have been detected in 1–4% of families negative for* BRCA* mutations [77]. Inherited mutations in the BRCA2-interacting protein, PALB2, are known to be associated with increased risks of breast, pancreatic, and, likely, ovarian cancer. Recently, Casadei and colleagues sequenced the coding sequences and flanking regulatory regions of* PALB2* from constitutional genomic DNA of 1,144 familial breast cancer* BRCA1/BRCA2*-negative patients.* PALB2* heterozygotes were four times more likely to have a male relative with breast cancer (*P* = 0.0003), six times more likely to have a relative with pancreatic cancer (*P* = 0.002), and 1.3-fold more likely to have a relative with ovarian cancer (*P* = 0.18) [10]. Overall, significantly less ovarian cancer is seen in* PALB2* families when compared with* BRCA1* and* BRCA2* families; therefore, it remains to be seen whether ovarian cancer risk is truly increased in individuals who are* PALB2* mutation carriers or not [77].

### 5.3. *CHEK2*

Furthermore, several groups have previously analyzed the role of* CHEK2* mutations in ovarian cancer cancerogenesis. Particularly, the missense variant of* CHEK2* I157T was significantly associated with ovarian cystadenomas, borderline ovarian tumors, and low-grade invasive cancers but not high-grade ovarian cancer [78]. In another study, Baysal and colleagues [79] identified del1100C and A252G variants of* CHEK2*, but since the differences in variant frequency were not statistically significant compared to controls, it was concluded that variations in* CHEK2* were not associated with ovarian cancer pathogenesis. A few years later, Krylova and colleagues [80] also failed to demonstrate an association between* CHEK2* 1100delC and ovarian cancer pathogenesis. Nevertheless, these analyses were mainly focused on some specific variants of* CHEK2* (del1100C, A252G, and I157T); therefore, mutations of other regions of* CHEK2* and their association with ovarian cancer pathogenesis still need to be investigated in detail [81].

### 5.4. Mre11 Complex

The Mre11 complex is composed of the proteins Mre11, NBS1, and RAD50 and represents a crucial component in the DNA repair process. Heikkinen and colleagues [82] screened 151 families with signs of hereditary breast and/or ovarian cancer for germline mutations in the* Mre11* complex genes. In this study, three potentially disease-related mutations were reported:* Mre11* 913C>T (Arg305Trp),* NBS1* 448C>T (Leu150Phe), and* RAD50* 687delT (stop codon at 234). These three mutations in the* Mre11* complex genes could also potentially be related to hereditary susceptibility to breast and ovarian cancer.

### 5.5. *BARD1*

Ratajska and colleagues [83] screened 109* BRCA1/2* negative high-risk breast and/or ovarian cancer patients from North-Eastern Poland for* BARD1* germline mutations and identified three different* BARD1* variants suspected to be pathogenic (c.1690C>T, p.Gln564X; c.1315- 2A>G; c.1977A>G). This study suggested that deleterious mutations in* BARD1* might be responsible for a certain proportion of familial breast and/or ovarian cancer.

## 6. Next-Generation Sequencing with Multigene Panels

Approximately 23% of ovarian carcinomas have been related to hereditary conditions and more than 15% of hereditary ovarian cancers are derived from a genetic condition unrelated to* BRCA* genes [3]. Particularly, several genes involved in the mechanism of hereditary ovarian tumorigenesis have been identified, but several mutations still remain unknown and cannot be detected by specific tests.

The identification of mutations in ovarian cancer susceptibility genes has a fundamental role both in the preventive setting, and after the diagnosis of ovarian cancer, in the selection of treatments. In healthy mutation carriers, the presence of one of these mutations may justify more intensive surveillance, chemopreventive approaches [84, 85], and/or prophylactic surgeries [86] that would not otherwise be justified by family history alone. In this particular setting, the candidates for genetic testing should be identified according to their personal and family history of ovarian cancer.

On the other hand, in already affected patients, the identification of a mutation in susceptibility genes may guide treatment decision-making by providing potential targets for biologic agents (i.e., PARP inhibitors) or by helping to select treatment strategies, that is, avoiding radiotherapy in patients with LFS [87]. Notably, a subgroup analysis of phase 2 trial data showed that olaparib maintenance therapy significantly prolongs PFS in patients with BRCA-mutated ovarian cancer (median, 11.2 versus 4.3 months; HR 0.18; 95% CI [0.10–0.31]; *P* < 0.0001) [88]. These results led to the accelerated approval of the PARP inhibitor, olaparib, by the European Medicines Agency (EMA) for the maintenance treatment of patients with platinum-sensitive, relapsed BRCA-mutated, high-grade serous epithelial ovarian, fallopian tube, or primary peritoneal cancer. More recently, the United States Food and Drug Administration (FDA) approved olaparib for the treatment of patients with ovarian cancer who have received three or more prior regimens and carry BRCA mutations. These approvals have crucial implications in the management of patients with high-grade serous tumors, which represent 75% of all epithelial ovarian cancers. In the near future, early and rapid genetic testing should be offered to every patient with these characteristics in order to provide the best therapeutic strategies.

Next-generation sequencing (NGS) technologies in recent years have facilitated an unprecedented capability to gain a better understanding of the genetic complexity of epithelial ovarian cancer. NGS technologies, based on massively parallel sequencing, offer several advantages over the previous techniques:

1. To analyze simultaneously multiple cancer susceptibility genes in one reaction.
2. To reduce the time required to complete the genetic analyses.
3. To screen additional loci at low additional costs.
4. Great sensitivity, specificity, and accuracy.

**Figure 5:**
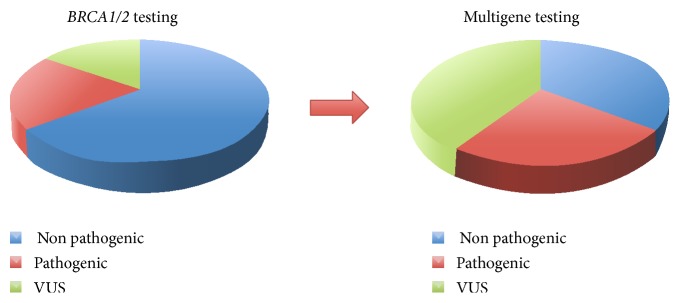
Proportion of nonpathogenic variants, pathogenic variants, and VUS in testing only* BRCA*1/2 genes or testing multiple genes simultaneously. The rate of VUS is expected to increase with the introduction of multigene panels.

Today, several multigene panels that include known ovarian cancer-associated loci have been introduced for the screening of germline mutations [89–91]. Nevertheless, there are significant challenges in interpreting and managing panel results. The main disadvantages of NGS techniques are listed below:

1. Although the overall cost continues to decrease, there are relevant initial costs associated with the technology such as sophisticated computer systems, bioinformatics tools, training of personnel, and, additionally, a significant time contribution.
2. Testing multiple genes simultaneously may identify an increasing number of variants of uncertain clinical significance (VUS), namely, mutations whose clinical significance has not yet been determined. Even for known high-risk genes, such as* BRCA1/2*, approximately 13% of families tested carry a “hard to interpret” variant [92], and this rate is expected to increase with the introduction of multigene panels (Figure 5) [93].
3. For several mutated genes, clinical management is complex and unclear. For example, LFS is characterized by a wide tumor spectrum and tumor risk in children; therefore,* TP53* testing should be carefully considered and all of the clinical implications of a positive test should be clearly explained before testing.
4. The simultaneous evaluation of multiple genes requires novel approaches to genetic counseling. Given the always increasing complexity of testing and results interpretation, a more articulate post-test counseling and longer-term clinical management are necessary.

Due to these challenges in interpreting and managing multigene panel results, much of this information is contained within the field of research and only in specialized centers should become standard of care. Mutational screening and genetic counseling by NGS should be centralized and carried on in specialized family cancer clinics, where patients and their families are appropriately informed of the limitations of these approaches, and then can be followed and managed by a multidisciplinary team over an extended period of time [94].

## 7. Conclusions

Ovarian cancer represents 3.7% of all female cancers. It is usually diagnosed in advanced stages with a poor prognosis and OS being the worst of all gynecologic malignancies [1, 2]. More than one-fifth of ovarian tumors have hereditary susceptibility [3], and in about 65–85% of cases the genetic abnormality is a germline mutation in* BRCA* genes. Nevertheless, several other suppressor genes and oncogenes have been associated with hereditary ovarian cancers, including mismatch repair (*MMR*) genes,* TP53*, and several genes involved in double-strand breaks repair. To date, at least 16 genes are known to be involved in the mechanism of hereditary ovarian tumorigenesis, but several mutations still remain unknown and cannot be detected by specific tests [12].

Defects in genes involved in the repair of double stranded breaks, other than* BRCA* 1 and 2, represent alternative mechanisms of hereditary ovarian carcinogenesis.* BRCA* negative tumors with a defect in the homologous recombination system express the BRCAness profile, a specific phenotype with features and behavior similar to* BRCA*-related cancers [8]. It is likely that these patients also might benefit from platinum-based therapies and/or PARP inhibition like BRCA mutation carriers do, but, to date, we still need to introduce validated tests into daily practice in order to identify patients with “BRCAness” profiles who carry mutations in genes such as* ATM*,* CHEK2*,* RAD51*,* BRIP1*, and* PALB2*.

In recent years, the development and implementation of NGS technologies have provided the opportunity to simultaneously analyze multiple cancer susceptibility genes, significantly increase the throughput, reduce the delay and costs, and optimize the molecular diagnosis of hereditary ovarian cancer. The identification of mutations in ovarian cancer susceptibility genes through multigene panels may result in more personalized cancer risk management with tailored clinical and radiological surveillance, chemopreventive approaches, and/or prophylactic surgeries [84–86]. On the other hand, for ovarian cancer patients, the identification of mutations may provide potential targets for biologic agents and help to guide treatment decision-making. Nevertheless, since there still are significant challenges in interpreting and managing panel results, much of this information still remains within the field of research and only in specialized centers should become standard of care. More intensive efforts to organize qualified family cancer clinics where the mutational screening and genetic counseling by NGS should be centralized and performed need to occur. Patients and families with mutations should be informed of the limitations of these approaches and then should be followed up and managed by a multidisciplinary team over an extended time period [94]. The centralization of genetic testing enables the improvement of access and quality of testing and allows for the creation of a more comprehensive database for research, guiding evidence-based management recommendations [89–91].

## Figures and Tables

**Table 1:** Different types of ovarian cancer with their clinicopathologic features and behavior.

	Type 1	Type 2
Prevalence	About 30%	About 70%

Histotype	Serous, endometrioid, mucinous, and clear-cell tumors	Serous, mixed malignant mesodermal tumors carcinosarcomas, and undifferentiated tumors

Grade	Low and borderline	High

Mutations	*PTEN, KRAS, BRAF, PIK3CA, ERBB2, CTNNB1, ARID1A*, *PPP2R1A*, and microsatellite instability	*TP53* *BRCA 1/2 *

Clinical behavior	Typically large cystic mass confined to the ovary, relatively indolent course	Diagnosed at advanced stages and aggressive behavior
